# Vaccination against *Onchocerca volvulus* induces IgG-mediated protective immunity dependent on neutrophils and complement

**DOI:** 10.1038/s41541-025-01267-x

**Published:** 2025-10-22

**Authors:** Nathan M. Ryan, Jessica A. Hess, Mohini Nakhale, Annabel A. Ferguson, William Stump, Sara Belko, Rachel Monane, Robert S. Pugliese, Nikolai Petrovsky, Benjamin L. Makepeace, Sean A. Gray, Darrick Carter, Sara Lustigman, David Abraham

**Affiliations:** 1 Department of Microbiology and Immunology, Sidney Kimmel Medical College, Thomas Jefferson University, Philadelphia, PA USA; 2 Department of Pathobiology, University of Pennsylvania School of Veterinary Medicine, Philadelphia, PA USA; 3 Health Design Lab, Sidney Kimmel Medical College, Thomas Jefferson University, Philadelphia, PA USA; 4 Vaxine Pty Ltd., Adelaide, SA Australia; 5 Institute of Infection, Veterinary & Ecological Sciences, University of Liverpool, Liverpool, UK; 6 PAI Life Sciences Inc., Seattle, WA USA; 7 Laboratory of Molecular Parasitology, Lindsey F. Kimball Research Institute, New York Blood Center, New York, NY USA

**Keywords:** Adjuvants, Antibodies, Immunological memory, Parasitic infection

## Abstract

Onchocerciasis remains a significant cause of morbidity and economic loss in sub-Saharan Africa. Despite the existence of effective therapeutics, a prophylactic vaccine targeting the etiologic agent, *Onchocerca volvulus*, is needed to control ongoing disease and transmission. Mice were vaccinated against *O. volvulus* with a fusion of the recombinant antigens *Ov*-103 and *Ov*-RAL-2 (*Ov*-FUS-1) with Advax-CpG adjuvant. Immunized mice developed protective immunity with the killing of third-stage larvae (L3) within 36 h of challenge infection. IgG from immunized mice passively transferred protective immunity to naïve mice, indicating that antigen-specific IgG mediated parasite elimination. Neutrophils were the most abundant subset of immune cells recruited to the parasite microenvironment in vivo, and treating mice with a granulocyte-depleting antibody resulted in the total loss of immune-mediated larval killing. Analysis of neutrophil gene expression revealed that both vaccination and the presence of *O. volvulus* larvae were capable of modulating neutrophil transcriptional activity. The mechanism by which antigen-specific IgG and neutrophils collaborated to kill L3 was independent of Fcγ receptors. However, the elimination of complement component C3 prevented vaccine-induced protection, which suggests these components may interact through the complement system. This study describes a vaccine-induced mechanism of protective immunity against *O. volvulus* L3 dependent on IgG, neutrophils, and complement, highlighting an effective collaboration between the innate and adaptive arms of the immune system to control *O. volvulus* infection.

## Introduction

Human onchocerciasis is a debilitating, neglected tropical disease caused by the parasitic filarial nematode *Onchocerca volvulus*. Infection occurs primarily in sub-Saharan Africa, where it is estimated that 21 million individuals are infected. Transmission of *O. volvulus* requires blackflies of the genus *Simulium* as the vector^1^. Infection is initiated when blackflies take a blood meal and transfer the *O. volvulus* infective third-stage larvae (L3) into the skin of the human host. The larvae molt into fourth-stage larvae (L4) and develop into male and female adult worms, which produce microfilariae. The death of microfilariae within the host releases endosymbiotic bacteria of the genus *Wolbachia*, inducing a pathological immune response. Depending on where microfilariae die, they can cause various complications, including intense itching, lymphadenopathy, impaired vision, or blindness^2^. High microfilarial load during childhood has also been associated with Nodding Syndrome, a disease characterized by paroxysmal head nodding, epileptic seizures, and mental decline^3^.

Current control of *O. volvulus* transmission is based primarily on mass drug administration (MDA) of ivermectin, which kills microfilariae and temporarily inhibits their release from adult worms, thereby reducing disease transmission. For ivermectin treatment to be successful, it must be administered annually for the entire 10–15-year reproductive lifespan of adult female worms^4,5^. Additional challenges in controlling onchocerciasis include lethal adverse reactions in areas of *Loa loa* co-infection^6^, possible resistance to ivermectin^7^, non-compliance with MDA programs^8^, and a lack of MDA approval for children under the age of five^9^. These complications form the rationale for developing additional strategies to aid in controlling and potentially eliminating onchocerciasis. A prophylactic vaccine that can be administered in addition to MDA efforts is critical to this effort. Based on mathematical modeling, a vaccine that could reduce the L3 burden by 50% would significantly reduce microfilarial loads and disease severity in infected individuals and reduce transmission in affected areas^10–12^. The effort to develop a prophylactic vaccine targeting *O. volvulus* has been led by The Onchocerciasis Vaccine for Africa (TOVA) initiative. This international consortium has primarily focused on identifying recombinant protein antigens and vaccine adjuvants capable of inducing protective immunity against infective *O. volvulus* L3. The consortium’s ultimate goal is to select an optimized formulation of the TOVA Vaccine (TOVAx) for broad distribution across endemic African communities^13^.

A mouse model was developed to study the biology and immunology of larval infections with *O. volvulus*. Diffusion chambers contain the larvae and allow the analysis of the in vivo parasite microenvironment^14^. Many antigens and adjuvants were screened in this model as vaccine candidates, which were evaluated based on their ability to induce protective immunity against infection^15,16^. These studies determined that *Ov*-103 and *Ov*-RAL-2 were the most effective antigens in protecting mice from *O. volvulus* challenge, and they were selected for further vaccine development. The original identification of these antigens was based on nematode specificity and reactivity with human serum antibodies from putatively and concomitantly immune individuals^17,18^. The adjuvanted antigens protected against infection when administered individually and as a co-administered bivalent vaccine^15–17^. Vaccination with *Ov*-103 and *Ov*-RAL-2 in genetically diverse Collaborative Cross Recombinant-inbred Intercrossed (CC-RIX) mice resulted in protective immunity against larval *O. volvulus* across various genetic backgrounds^19^. Vaccines composed of *Ov*-103 and *Ov*-RAL-2 or their homologs have been effective at inducing protective immunity in cows to *Onchocerca ocheng*i^20^, in non-human primates to *O. volvulus*^21^, and in jirds to *Brugia malayi*^22^. A recombinant fusion protein composed of these two antigens (*Ov*-FUS-1) induced an accelerated killing of the larvae compared to co-administration of the two individual antigens. Evaluation of adjuvants for efficacy with *Ov*-FUS-1 in mice revealed that adding Advax-CpG adjuvant resulted in a more durable protective immune response relative to alum. In contrast, the addition of AlT4 did not induce significant protection. Serum collected from both mice and NHPs immunized with *Ov*-FUS-1/Advax-CpG could transfer protection to naïve mice, suggesting the protective immune response induced by vaccination was mediated by antigen-specific antibody^21^. Furthermore, a predictive immunoinformatic study of *Ov*-103 and *Ov*-RAL-2 demonstrated that the antigens have favorable safety profiles and multiple immunogenic epitopes, thereby providing evidence for the potential clinical success of the *Ov*-FUS-1 based *O. volvulus* vaccine^23^.

Vaccine development has long been conducted through empirical observation rather than strategic design based on immunological principles^24^. An immunology-based vaccine development approach is especially important when optimizing vaccines against complex pathogens like helminths. In addition, knowledge of the vaccine’s mode of action would allow clinical monitoring of vaccine efficacy through appropriately selected biomarkers. The present study aimed to identify the mechanism whereby elements of the innate and adaptive immune responses control *O. volvulus* L3 infection in mice immunized with the *Ov*-FUS-1/Advax-CpG vaccine. Antigen-specific IgG and complement factor C3 were evaluated for their role in the immune-mediated killing of parasites after vaccination. Neutrophils were assessed to determine if they participated in the vaccine-induced protective immune response, interacted directly with antigen-specific IgG, and if the vaccination process transcriptionally altered them. The cellular and humoral components induced by vaccination were defined, and their collaboration to protect against a complex metazoan parasite was elucidated.

## Results

### IgG from immunized mice transfers protection against *O. volvulus* L3 to naïve mice

**Fig. 1:**
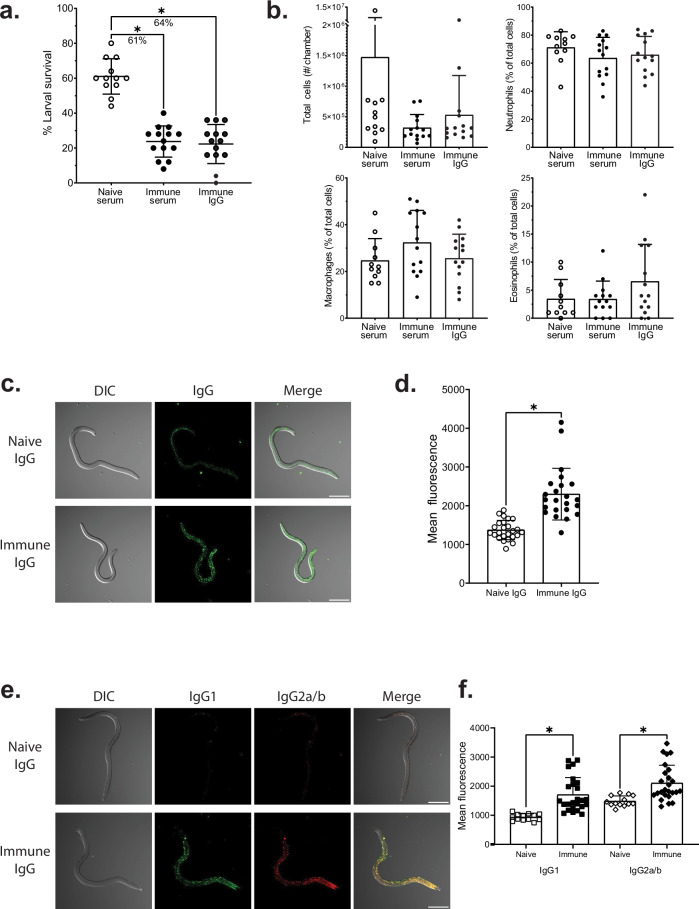
IgG purified from immunized mouse serum protects mice against challenge with *O. volvulus* L3. **a**, **b** IgG was purified from mice vaccinated with *Ov*-FUS-1/Advax-CpG. Mice received either naïve serum, immune serum, or purified immune IgG and were challenged with *O. volvulus* L3 in diffusion chambers. Surviving larvae and diffusion chamber contents were collected seven days post-challenge. **a** Survival of *O. volvulus* L3 in diffusion chambers. Data are shown as mean percent larval survival. Error bars represent standard deviation. Percent reduction in larval survival between each group and control animals is indicated under each bracket. **b** Total cells and differential cell counts of neutrophils, macrophages, and eosinophils as a percentage of total cells in diffusion chambers. Data are shown as mean total cell counts or percentage of total cells. Points representing individual mice from two replicate experiments with error bars indicating standard deviations. **c**–**f** *O. volvulus* L3 were incubated with either naïve mouse IgG or purified immune IgG. **c** Representative images from L3 incubated with naïve IgG (top) and immune IgG (bottom) and stained with anti-mouse IgG fluorescent antibody. **d** Quantification of mean fluorescence for total mouse IgG. **e** Representative images from L3 incubated with naïve IgG (top) and immune IgG (bottom) and stained with fluorescent anti-mouse IgG1 and combined anti-IgG2a and IgG2b (IgG2a/b) antibodies. **f** Quantification of mean fluorescence for IgG1 and IgG2a/b. **c** and **e** Scale bar: 200 µm. **d** and **f** Data are shown as mean fluorescence intensity over the area of individual worms, indicated by points. Error bars represent standard deviations. *p value < 0.05, indicating statistically significant differences when comparing groups.

Previous studies demonstrated that serum from mice immunized with *Ov*-FUS-1/Advax-CpG could transfer protection against *O. volvulus* L3 to naïve mice^21^. To test the hypothesis that antigen-specific IgG is the active component in serum responsible for protection, IgG was purified from immunized mouse serum and transferred into naïve BALB/cByJ mice. The mice were challenged with L3, and the worms recovered seven days later. Mean larval survival was 61% in mice treated with naïve serum, which was significantly reduced to 24% with whole immune serum and 22% with purified immune IgG. These results indicate that IgG from immunized mouse serum is sufficient for transferring protection against *O. volvulus* L3 to naïve mice (Fig. 1a).

Total cell counts within the diffusion chambers did not differ significantly between mice regardless of treatment. Neutrophils, macrophages, and eosinophils were measured within the diffusion chambers, with similar proportions when comparing the three treatment groups. Neutrophils were the most abundant cell type ranging from a mean of 64–72% of total cells (Fig. 1b).

To determine whether the purified immune IgG bound specifically to the surface of *O. volvulus*, L3 were incubated in either naïve mouse IgG or purified immune IgG and then stained with fluorescent anti-mouse IgG secondary antibody. Purified immune IgG bound to the L3 surface in a punctate signal distributed along the entire worm, with no apparent specificity for particular regions or structures (Fig. 1c). L3 incubated in purified immune IgG had a significant increase in fluorescence intensity compared to incubation in naïve IgG, demonstrating immune IgG specificity for the L3 surface (Fig. 1d). Larvae were also stained with fluorescent antibodies against either IgG1 or combined IgG2a and IgG2b (IgG2a/b). L3 incubated in purified immune IgG stained positively for both IgG1 and IgG2a/b with punctate staining equally distributed across the surface (Fig. 1e). The fluorescence intensities marking either IgG1 or IgG2a/b were significantly increased on larvae incubated in immune IgG relative to naïve IgG, demonstrating both IgG subclasses bind specifically to the larval surface (Fig. 1f).

### Vaccination induces *O. volvulus* L3 killing between 18 and 36 hours post-challenge

**Fig. 2:**
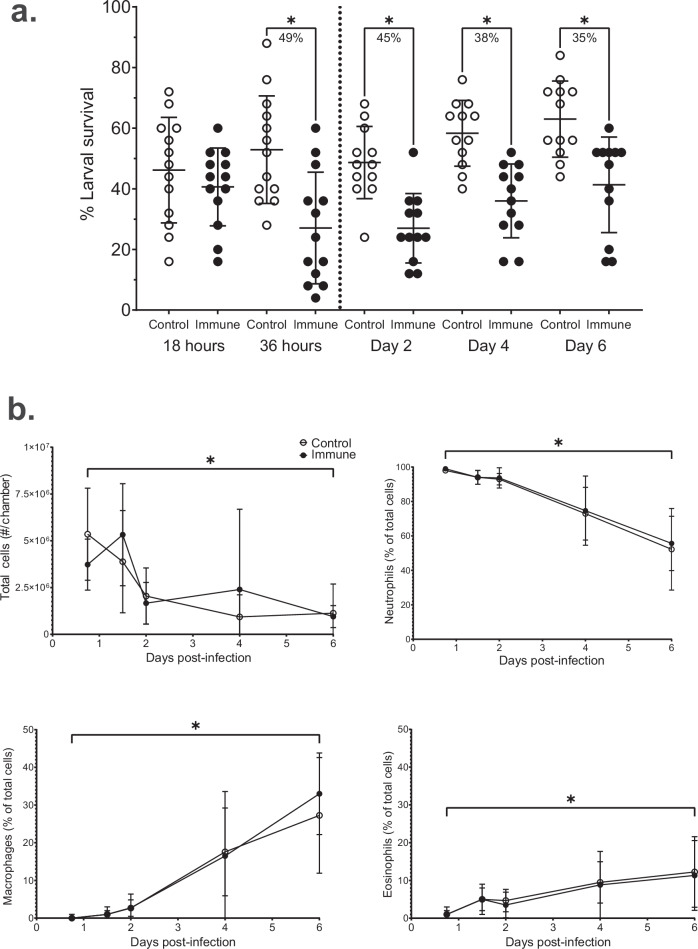
Immunization with *Ov*-FUS-1/Advax-CpG induces *O. volvulus* larval killing between 18 and 36 hours post-challenge in mice. BALB/cByJ mice were immunized with *Ov*-FUS-1/Advax-CpG and challenged with *O. volvulus* L3 in diffusion chambers. Recovery of diffusion chambers occurred 18 h, 36 h, two days, four days, and six days post-challenge. **a** Survival of *O. volvulus* L3 in diffusion chambers. Data are shown as mean percent larval survival with individual mice from two replicate experiments represented as points. Error bars represent standard deviation. The percent reduction for significant differences in larval survival between each group and its respective control is indicated under each bracket. **b** Total cells and differential cell counts of neutrophils, macrophages, and eosinophils as a percentage of total cells in diffusion chambers. Data are shown as mean total cell counts or percentage of total cells at each time point. Error bars indicate standard deviations. *p < 0.05, indicating statistically significant differences when comparing groups.

In previous experiments evaluating protection induced by immunization with *Ov*-FUS-1/Advax-CpG, challenge larvae were recovered seven days post-challenge^21^. To determine the larval stage targeted by the protective immune response following vaccination, mice were immunized and challenged with L3, followed by recovery of challenge larvae after 18 h, 36 h, two days, four days, and six days. At the 18-h time point, mean larval survival was equivalent in control and immunized mice. At 36 h, mean larval survival was 53% in control mice and 27% in immunized mice, corresponding to a significant 49% reduction (Fig. 2a). Mean larval survival in immunized mice was significantly reduced relative to controls at each remaining time point, with no increase in the magnitude of larval killing. When the study terminated six days post-challenge, there was a reduction of 35% in mean larval survival between control and immunized mice (Fig. 2a). These observations demonstrate the vaccine-induced immune response kills *O. volvulus* L3 between 18 and 36 h post challenge.

Total cell infiltration into the diffusion chambers was equivalent between control and immunized mice regardless of time point. The total number of cells decreased significantly between 18 h and 6 days in both control and immunized mice (Fig. 2b). At 18 h post challenge, on average, 99% of cells in the diffusion chambers of immunized mice were neutrophils, and 1% were eosinophils, with no measurable number of macrophages. At 36 h post challenge, the proportion of neutrophils in the diffusion chambers of immunized mice decreased slightly to a mean of 94%, while macrophages constituted 1% and eosinophils increased to 5%. These proportions were similar in control mice at both time points (Fig. 2b). Throughout the six-day challenge, the proportion of neutrophils declined as macrophages and eosinophils increased. By six days post-challenge, neutrophils remained the dominant cell type in diffusion chambers from immunized mice, having decreased to an average of 56%, with 33% macrophages and 11% eosinophils. Similar proportions were measured in control mice (Fig. 2b).

The role of L3 in cell recruitment was evaluated by implanting diffusion chambers with and without larvae for 18 and 36 h in control and immunized mice. Total cell counts and percentages of neutrophils, macrophages, and eosinophils were equivalent regardless of the presence or absence of *O. volvulus* L3 at both time points (Supplementary Table 1). These observations demonstrate that neutrophils are the dominant cell type in diffusion chambers implanted in control and immunized mice at the time point immediately before and after the killing process occurs, and that the recruitment is independent of the presence of larvae.

### Neutrophils are required for the killing of challenge larvae following vaccination

**Fig. 3:**
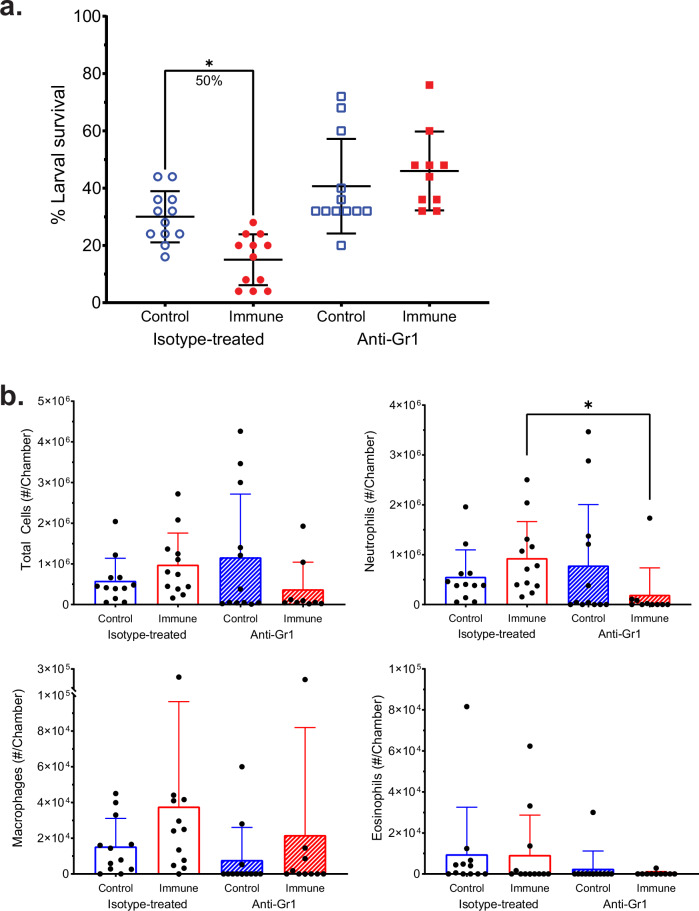
Vaccine-induced protection against *O. volvulus* L3 requires neutrophils in mice. BALB/cByJ mice were immunized with *Ov*-FUS-1/Advax-CpG and challenged with *O. volvulus* L3 in diffusion chambers. Mice were treated with either anti-Gr1 antibody or isotype control, and diffusion chambers were recovered 48 h post-challenge. **a** Survival of *O. volvulus* L3 in diffusion chambers. Data are shown as mean percent larval survival with individual mice represented as points. Error bars represent standard deviation. The percent reduction for significant differences in larval survival between each group and its respective control is indicated under each bracket. **b** Total number of cells, neutrophils, macrophages, and eosinophils in diffusion chambers. Data are shown as mean total cell counts with error bars representing standard deviations. Data points represent individual mice, and error bars indicate standard deviations. *p value < 0.05, indicating statistically significant differences when comparing groups.

Granulocytes were depleted from immunized mice using a Gr1-specific antibody to investigate their role in protective immunity. Diffusion chambers were recovered 48 h post challenge, and larval survival was measured. In the isotype control-treated group, mean larval survival was 30% in control mice and 15% in immunized mice, corresponding to a significant 50% reduction in larval survival. In the anti-Gr1-treated group, there was no significant difference in mean larval survival between control and immunized mice (Fig. 3a).

Total cell counts in the diffusion chambers recovered from isotype- and anti-Gr1-treated mice were equivalent, regardless of whether they were immunized (Fig. 3b). The number of neutrophils was significantly reduced, while macrophages and eosinophils were equivalent in anti-Gr1-treated mice relative to isotype-treated mice (Fig. 3b). The lack of protection in immunized mice after treatment with anti-Gr1 antibody indicates that protection against *O. volvulus* larval challenge is dependent on the presence of neutrophils in the parasite microenvironment.

### Immunization of mice results in altered transcription of neutrophil gene sets

Neutrophils are the dominant cell type in the parasite microenvironment at the time *O. volvulus* L3 are killed in both control and immune mice (Fig. 2b), yet they are required for killing to occur (Fig. 3a). These observations suggest that functional rather than quantitative differences in neutrophils in immunized mice may explain their increased L3 killing capacity. Degranulation is one potential neutrophil function that may account for parasite killing in immunized mice. However, concentrations of myeloperoxidase (MPO) and neutrophil elastase (NE) measured in the diffusion chamber fluid at 18 and 36 h post challenge were equivalent between control and immunized mice.

The lack of differential release of key neutrophil granule products led a broader approach to investigating functional differences between neutrophils of control and immunized mice. The transcriptional profiles of cells from diffusion chambers recovered at 18 and 36 h post challenge, with or without L3, were analyzed by bulk RNA sequencing (RNA-seq) to determine whether immunization induced differential gene expression that may be associated with larval killing. Since neutrophils constituted 96–99% of cells within diffusion chambers across all groups, the samples were considered pure neutrophil populations for analysis. Differences in global gene expression between all samples were analyzed by hierarchical clustering of Euclidian distances. Samples from 18 and 36 h post challenge were distinguishable by hierarchical clustering, while any further distinction between immunized and control samples or the presence or absence of larvae was not apparent (Supplementary Fig. 1). This analysis suggests that the duration of diffusion chamber implantation had the most significant impact on neutrophil global gene expression.

**Fig. 4:**
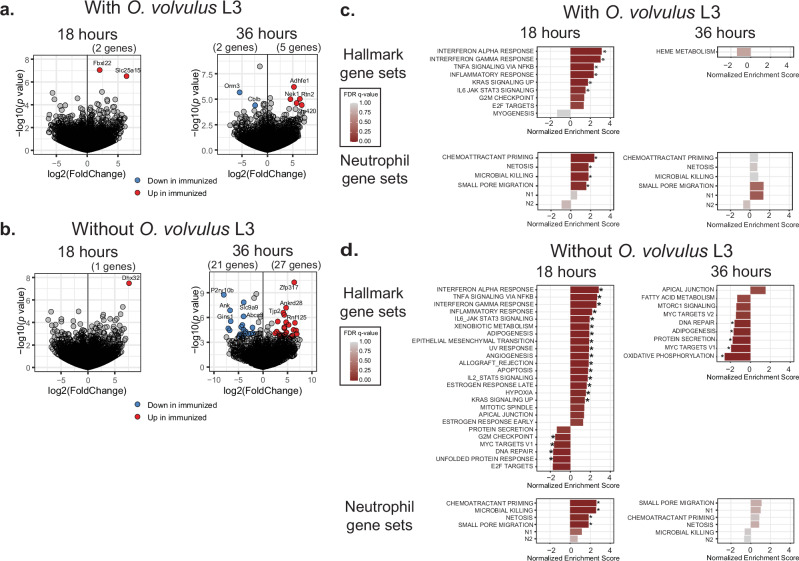
Immunization induces differential gene expression in neutrophils recovered from diffusion chambers 18 and 36 hours post-challenge. BALB/cByJ mice were immunized and challenged. Recovery of diffusion chambers occurred 18 and 36 h post-challenge, and cells were collected for bulk RNA-Seq. Volcano plots for differential gene expression of neutrophils from diffusion chambers with *O. volvulus* L3 **a** or without **b**. Scatter plot of -Log10(*p*-value) (vertical axis) by Log2(fold-change) of immunized versus control samples (horizontal axis) for each gene. Red-colored points indicate a fold-change greater than 2, with an adjusted *p*-value less than 0.05, and blue points indicate a fold-change less than –2, with an adjusted *p*-value < 0.05. Data are presented for neutrophils recovered 18 h (left) and 36 h (right) post-challenge. Plots of normalized enrichment scores (horizontal axis) for all identified gene sets with a *p*-value < 0.05 after implantation of diffusion chambers with *O. volvulus* larvae **c** or without **d**. Data are presented for Hallmark gene sets (top) and neutrophil gene sets (bottom) for neutrophils recovered 18 h (left) and 36 h (right) post-challenge. The color of the bars indicates the magnitude of FDR adjusted *p*-value, * FDR *p*-value < 0.05. Error bars represent standard deviations. *p value < 0.05, indicating statistically significant differences when comparing groups.

To examine whether prior immunization was associated with differential expression of any genes, we analyzed the fold-change expression differences via DESeq2 between immunized and control mice, sub-divided into four analysis groups according to the presence or absence of larvae and 18 or 36 h post challenge. In the two groups with larval challenge, there were surprisingly few differentially expressed genes by the threshold of Log2 fold-change greater than 2 or less than –2. Among these, two genes were upregulated in the immunized group 18 h post challenge, and at the 36-h time point, five genes were upregulated and two downregulated compared to controls (Fig. 4a, Supplementary File 1). In the comparison groups without larvae, only a single gene was upregulated at the 18-h time point, while the 36-h time point had 27 upregulated and 21 downregulated genes (Fig. 4b, Supplementary File 1). These results demonstrate that immunization of mice alters the gene expression in neutrophils and that the presence of larvae in the neutrophil microenvironment diminishes the number of upregulated and downregulated genes expressed in neutrophils of immunized mice.

Differential gene set enrichment analysis (GSEA) was performed to assess the functional characteristics predicted by gene expression of our treatment groups. Alterations in biological functions were assessed by asking if any hallmark gene sets within the MSigDB were coordinately regulated in our four comparison groups^25^. Six hallmark gene sets had significant positive enrichment in the immunized 18-h group with larval challenge (FDR *p*-value < 0.05) (Fig. 4c). Among these, the foremost enriched pathways were interferon (IFN)-α, IFN-γ, tumor necrosis factor-alpha (TNF-α), IL-6, and inflammatory response. Notably, there were no hallmark gene sets that were significantly downregulated. This analysis suggests that immunization enhanced the activation of IFN and inflammatory pathways in neutrophils at 18 h post challenge. A dataset of six neutrophil-type-specific gene signatures compiled from previous studies was used to further probe the functional state of the neutrophils within the diffusion chambers^26^ (Supplementary File 2). GSEA at the 18-h time point in diffusion chambers containing larvae identified four significantly over-represented gene sets associated with immunization (Fig. 4c). The enriched gene sets included chemoattractant priming, neutrophil extracellular trap formation (NETosis), microbial killing, and small pore migration^26–29^. These gene sets were also enriched with immunization in the control groups without larvae in the diffusion chambers (Fig. 4d). The GSEA results at 36 h post challenge had no significantly enriched hallmark or neutrophil-functional gene sets with larval implantation (Fig. 4c). Similarly, there were no gene sets with positive enrichment scores in the control groups without larval challenge at 36 h, and six negatively enriched gene sets from the hallmark gene sets (Fig. 4d). Interestingly, differences in neutrophil functional gene sets in immunized mice were observed at the 18-h time point, while the larval killing was not apparent until 36 h post challenge (Fig. 2a), suggesting that neutrophils develop functional transcriptional differences at the 18-h time point before carrying out their larval-killing function.

### Vaccine-induced killing of *O. volvulus* larvae is independent of Fcγ receptors

**Fig. 5:**
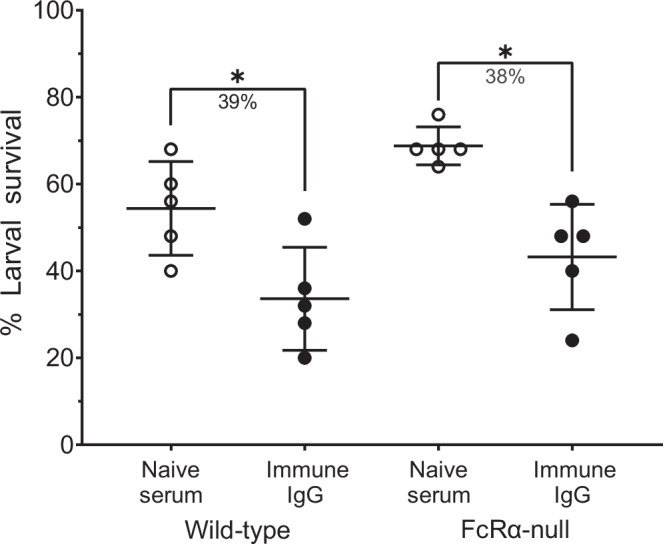
Fcγ receptors are not required for IgG-mediated protection against *O. volvulus* L3. C57BL/6J wild-type and FcRα-null mice (deficient in all FcγRs) received either naïve serum or purified immune IgG. Diffusion chambers were recovered seven days post-challenge. Data are shown as mean percent larval survival, with individual mice represented as points and error bars representing standard deviation. Percent reduction for significant differences in larval survival between each group and its respective control is indicated under each bracket. *p value < 0.05, indicating statistically significant differences when comparing groups.

To investigate whether IgG and neutrophils interact directly to kill *O. volvulus* larvae via FcγRs, mice deficient in all FcγRs (FcRα-null)^30^ and C57BL/6J wild-type mice received either naïve serum or purified immune IgG and larval survival was measured seven days post-challenge. In wild-type mice, mean larval survival was 54% when receiving naïve serum and 34% with immune IgG, corresponding to a significant 39% reduction. In FcRα-null mice, mean larval survival was 69% when receiving naïve serum and 43% with immune IgG, corresponding to a significant 38% reduction (Fig. 5). Total cell numbers and the proportion of neutrophils, macrophages, and eosinophils within the diffusion chambers were quantified; however, no consistent differences were observed when comparing any of the groups (Supplementary Fig. 2). This experiment was replicated in a second cohort with the same results. Equivalent reductions in mean larval survival between wild-type and FcγR-deficient FcRα-null mice demonstrate that IgG-mediated killing of *O. volvulus* L3 occurs independently of FcγRs.

### Complement component C3 is required for killing of challenge larvae following vaccination

**Fig. 6:**
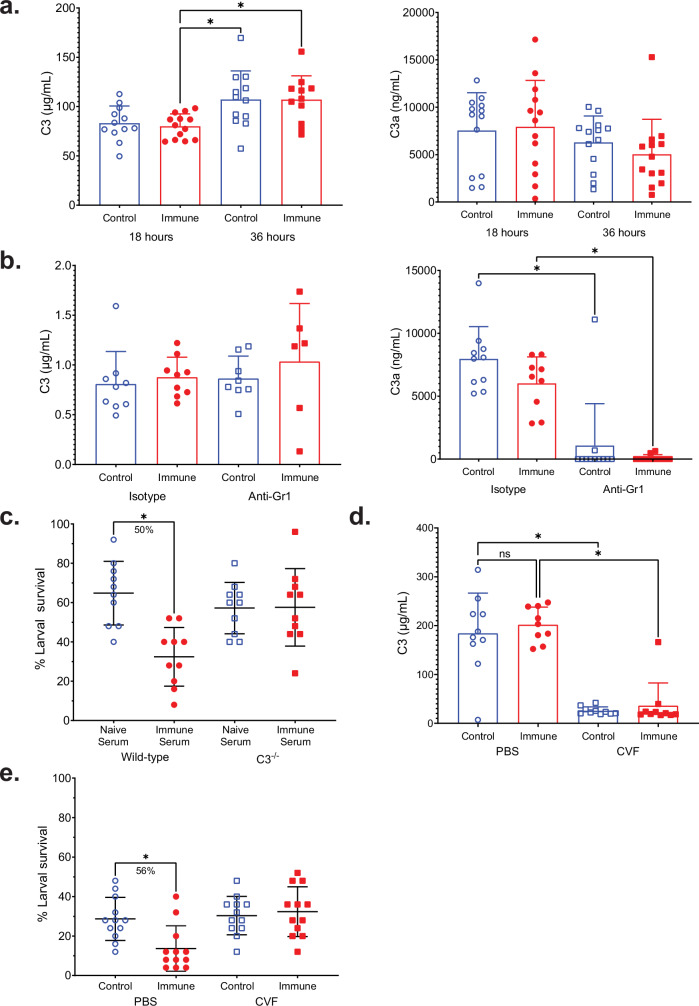
Complement component C3 is required for vaccine-induced protection against *O. volvulus* L3. **a** BALB/cByJ mice received either vaccination or control injection before challenge. C3 (left) and C3a (right) were measured in diffusion chamber fluid recovered at 18 and 36 h post-challenge. Data are shown as concentrations of C3 or C3a in the diffusion chamber. **b** BALB/cByJ mice were immunized, challenged, and treated with either anti-Gr1 antibody or isotype control. Data represent C3 (left) and C3a (right) concentrations in the diffusion chambers 48 h post-challenge. **c** Survival of *O. volvulus* L3 in wild-type and C3^–/–^ mice treated with either naïve or immune serum recovered seven days after diffusion chamber challenge. Data are shown as mean percent larval survival. **d**–**f** BALB/cByJ mice were immunized, challenged, and treated with either cobra venom factor (CVF) or PBS control. **d** C3 levels in diffusion chamber fluid were measured by ELISA 48 h post-challenge. Data are shown as mean optical density (OD) with points. **e** Survival of *O. volvulus* L3 in diffusion chambers. Data are shown as mean percent larval survival. **a**–**e** Data points represent measurements for individual mice from two replicate experiments, while error bars indicate standard deviations. **c** and **e** Percent reduction for significant differences in larval survival between each group and its respective control is indicated under each bracket. *p value < 0.05, indicating statistically significant differences when comparing groups.

The binding of IgG to antigens on the surface of target pathogens has been shown to induce the complement cascade, resulting in the recruitment of immune cells such as neutrophils^31^. To assess the role of complement in vaccine-induced protection, the concentration of C3 was measured in diffusion chamber fluid collected at 18 and 36 h post challenge. There was a significant increase in C3 levels in both control and immunized mice at 36 h, compared to immunized mice at 18 h, with no differences in C3a levels between any of the groups (Fig. 6a). In control and immunized mice treated with either anti-Gr1 antibody or isotype control, C3 in the diffusion chamber fluid was consistent across all groups. However, C3a was significantly reduced in anti-Gr1-treated mice compared to isotype-treated controls. The reduction of C3a in anti-Gr1-treated mice suggests that neutrophils play a functional role in regulating complement activity, specifically C3a production (Fig. 6b).

Wild-type C57BL/6J and C3^–/–^ mice received the passive transfer of either naïve or immune serum, and larval survival was measured seven days post-challenge. In the wild-type mice, mean larval survival was 65% in mice that received naïve serum and 32% in those receiving immune serum, resulting in a significant 50% reduction. In C3^–/–^ mice, mean larval survival was equivalent in mice that received naïve or immune serum (Fig. 6c). Total cells and the proportion of neutrophils, macrophages, and eosinophils within the diffusion chambers were equivalent across all groups (Supplementary Fig. 3a). The loss of a significant reduction in mean larval survival in C3^–/–^ mice that received immune serum demonstrates that C3 is required for IgG-mediated protection.

To confirm the requirement for C3 in the vaccine-induced protective immune response, immunized BALB/cByJ mice were treated with CVF to deplete circulating C3 and then challenged with L3. Forty-eight hours post-challenge, larval survival was evaluated. The depletion of C3 was confirmed in diffusion chamber fluid by ELISA, which showed a significant reduction in C3 levels in CVF-treated mice compared to PBS-treated mice (Fig. 6d). In PBS-treated mice, mean larval survival was 29% in control mice and 14% in immunized mice, corresponding to a 56% reduction. In CVF-treated mice, mean larval survival was equivalent between control and immunized mice (Fig. 6e). Total cells and the proportion of neutrophils, macrophages, and eosinophils were measured within the diffusion chambers; however, there were no consistent differences between any of the groups (Supplementary Fig. 3b). These data demonstrate that complement is required to kill challenge parasites following immunization and passive transfer of immune serum.

## Discussion

The present study describes the dynamics and key components in the mechanism of protective immunity induced by vaccination with the *Ov*-FUS-1/Advax-CpG vaccine. Based on the data presented, it was concluded that killing occurs between 18 and 36 h after challenge with *O. volvulus* L3. The mechanism of this protective immune response is mediated by vaccine-induced IgG and dependent on C3 and transcriptionally distinct neutrophils, which function independently of FcγRs.

Vaccinating mice and NHPs with *Ov*-FUS-1/Advax-CpG was previously shown to induce high serum titers of antigen-specific IgG. Transfer of immune serum from both mice and NHPs to naïve mice resulted in a significant decrease in larval survival, suggesting that protection is mediated by vaccine-induced serum IgG^21^. The transfer of protective immunity to naïve mice using purified IgG from the serum of mice immunized with *Ov*-FUS-1/Advax-CpG demonstrates that IgG mediates the vaccine-induced protection mechanism. These findings are consistent with previous studies that showed AID^–/–^ mice vaccinated with *Ov*-103 or *Ov*-RAL-2 failed to establish protection against *O. volvulus* challenge infection^17^. IgG and other antibody classes are protective in secondary infections with the nematodes *Strongyloides stercoralis*^32,33^ and *Heligmosomoides polygyrus*^34^. Previous studies demonstrated that protective immunity generated by immunizing mice with irradiated *O. volvulus* L3 was dependent on IgE and eosinophils^35^. However, mice immunized with *Ov*-FUS-1/Advax-CpG did not generate a measurable IgE serum titer^21^. Therefore, the protection mechanism described presently differs from that induced by irradiated larvae. An IgG-mediated immune response and concurrent lack of IgE may result in a more favorable safety profile, as an IgE-mediated response to vaccination has been associated with adverse reactions^36^.

The antigen-specific IgG bound to the surface of *O. volvulus* L3 produced punctate signal across the surface of the larvae, similar to previous studies that investigated the localization of *Ov*-103 and *Ov*-RAL-2^22,37^. Staining for mouse IgG1 and IgG2a/b indicated that both subclasses bind to the surface of *O. volvulus* in a similar punctate manner, with no apparent specificity for individual surface structures. A balanced response of both IgG1 and IgG2a/b may be essential for eliminating pathogens while limiting immunopathology^38^. The importance of a balanced IgG response has been demonstrated in *Mastomys coucha* immunized with the recombinant *Wuchereria bancrofti* antigen rWbGST, where increased serum IgG1 and IgG2a was associated with cross-protection against the filarial parasite *B. malayi*^39^. A balanced antibody response was associated with improved efficacy and durability of the vaccine-induced protective immune response against *O. volvulus* in CC-RIX mice^19^, cows^20^, inbred mice and NHPs^21^.

Recovery of surviving challenge larvae at 18 and 36 h post challenge from immunized mice demonstrated that killing occurred between these two time points. Furthermore, no additional killing was observed up to six days post-challenge, and in a previous study, no additional killing occurred up to 3-weeks post-challenge^21^. Killing of *O. volvulus* larvae before 36 h post challenge demonstrates that the target of the protective immune response is the L3 stage, as the larvae begin molting into L4 three days after diffusion chambers are implanted in both primate and rodent hosts^14^. The antigens *Ov*-103 and *Ov*-RAL-2 have not been detected on the surface of L4^18,40^, and immunization of mice with irradiated *O. volvulus* L4s did not protect against L3 challenge^41^, suggesting that protective immunity is stage-specific. Susceptibility may return in subsequent developmental stages when these antigens become expressed again in adults and microfilariae^17,22^. The efficacy of a vaccine composed of *Ov*-103 and *Ov*-RAL-2 against adults and microfilariae of *O. ochengi* has been demonstrated in immunized cattle. Immunized cows had a 35% reduction in adult female burden and a 70% reduction in skin microfilariae following natural infection with *O. ochengi*^20^. In addition to increased vaccine efficacy by targeting later stages, eliminating microfilariae may reduce *O. volvulus* transmission.

The most abundant cell type within the diffusion chambers were neutrophils, regardless of the time point or whether mice were immunized. After *O. volvulus* killing occurred in immunized mice by 36 h, the proportion of neutrophils began to decline and was replaced with macrophages and eosinophils. This trend continued over the six-day course of the challenge and likely represents a transition away from IFN and inflammation-associated protective responses towards wound repair and clearance of dead larvae. This hypothesis is supported by the observation that macrophages and eosinophils respond to dead *O. volvulus* microfilariae in human lymph nodes^42^.

The depletion of neutrophils using an anti-Gr1 antibody led to the loss of significant larval killing in the immunized mice. The antigen Gr1 targets the Ly6C/Ly6G surface protein complex, which is expressed by multiple myeloid cell subsets, including neutrophils, eosinophils, and macrophages^43^. However, the numbers of eosinophils and macrophages in the diffusion chambers remained unchanged following anti-Gr1 treatment, demonstrating that neutrophils were required in immunized mice for protection against *O. volvulus* L3 challenge. Neutrophils were shown to interact with serum antibodies from infected and putatively immune humans, resulting in the in vitro killing of *O. volvulus* L3 through antibody-dependent cellular cytotoxicity (ADCC)^44^. Neutrophils have also been shown to collaborate with human monospecific anti-*Ov*-103 antibodies, but not anti-*Ov*-RAL-2 antibodies, to inhibit *O. volvulus* L3 molting in vitro^17^.

The number of neutrophils and the levels of MPO and NE were equivalent in both control and immunized mice, suggesting neutrophil degranulation alone is insufficient to mediate protection. However, neutrophils were determined to be critical to vaccine-induced protection. It was hypothesized that immunization induces differential neutrophil gene expression, altering their function and thus illuminating their role in protective immunity. Neutrophils from diffusion chambers with *O. volvulus* L3 implanted in immunized mice were enriched for several gene sets associated with IFN signaling and neutrophil effector functions. Without L3, neutrophils from immunized mice had increased transcription for the same gene sets enriched with L3 and additional enrichment in several metabolic and homeostatic pathways. These observations suggest that the vaccine alters neutrophil gene expression, and *O. volvulus* L3 exposure downregulates several pathways associated with metabolic and homeostatic processes. The ability of vaccine-primed neutrophils to maintain enrichment of IFN, inflammatory, and effector function gene sets in the presence of *O. volvulus* may be critical in the protection mechanism. Notably, Advax-CpG adjuvant, which enhances *Ov*-FUS-1 protection^21^, has been shown to induce T helper type 1 (Th1) T-cell IFN-γ recall responses to co-administered vaccine antigens^45^. Alum, which failed to achieve similar levels of *Ov*-FUS-1 protection, imparts a strong T helper type 2 (Th2) bias to T-cell recall responses^21^. Interferon pathways can amplify complement activation^46^ and induce IgG switching from non-complement active to complement active isotypes^47,48^. Hence, the combination of immune IgG, complement, and IFN-polarized neutrophils may provide the optimal environment for larval killing.

The enhanced transcriptional state of neutrophils subsided 18 h later, as gene set enrichment was effectively equivalent across all groups by 36 h post challenge, except for a few metabolic pathways that were downregulated in immunized mice without larvae. The transition in neutrophils from upregulated inflammatory pathways and effector function to control-level transcription between 18 and 36 h post challenge corresponds with time points pre- and post-killing of *O. volvulus* L3 in immunized mice. These results suggest that in mice immunized with *Ov*-FUS-1/Advax-CpG, there is a rapid neutrophil transcriptional response, followed by a quiescent state after these early activation and effector functions occur. Vaccine-induced neutrophil priming has been previously described in humans following immunization against tuberculosis, where neutrophils were shown to undergo epigenetic changes that resulted in increased activation markers and antimicrobial function three months after vaccination^49^.

The enrichment of IFN and inflammatory signaling and neutrophil effector pathways prior to *O. volvulus* killing may indicate specific protective mechanisms. The upregulation of TNF-α and IL-6 signaling pathways enhances complement activation and neutrophil degranulation^50,51^. The observation that IgG from immunized mice can passively transfer protective immunity raises the question of whether immune IgG alone induces similar transcriptional changes in naïve neutrophils.

The conclusion that vaccine-induced IgG, C3, and neutrophils are required to kill *O. volvulus* L3 in mice following immunization raises the question of how these components interact. Previous studies demonstrated that protective immunity induced by vaccination with alum-adjuvanted *Ov*-103 and *Ov*-RAL-2 was dependent on cellular contact with *O. volvulus* L3^17^. This observation led to the hypothesis that neutrophils interact with the Fc portion of IgG via FcγRs to kill *O. volvulus* larvae through an ADCC-dependent mechanism. However, FcRα-null mice that received immune IgG from *Ov*-FUS-1/Advax-CpG immunized mice were protected from *O. volvulus* L3 challenge, demonstrating that FcγRs are not necessary for the vaccine-induced killing mechanism. These differences may reflect the distinct mechanisms by which the alum and Advax-CpG adjuvants shape the adaptive immune response even to the same antigens.

The complement pathway presents an alternative mechanism through which cells and antibodies can collaborate to kill *O. volvulus*. The importance of the complement system during *O. volvulus* infection is demonstrated by the fact that microfilariae have evolved to evade complement by binding host factors that inactivate C3b^52^. The present study shows that C3 is required for killing *O. volvulus* in mice following either vaccination or passive transfer of immune serum from *Ov*-FUS-1/Advax-CpG immunized mice. These data implicate the complement system as a potential link between IgG and neutrophils. The binding of IgG to complement component 1q (C1q) results in the initiation of the complement cascade and hydrolysis of C3 into C3a and C3b^53^. C3a and C3b are important mediators of immune protection and can activate and recruit neutrophils by binding receptors on their surface^54,55^.

C3 and C3a levels in diffusion chambers were equal in control and immunized mice, with C3a levels being significantly reduced following anti-Gr1 treatment. This phenomenon suggests that neutrophils may play an important role in the maintenance of the complement system. Neutrophils release properdin, either in secondary granules or with NETs, which stabilizes the C3 convertase of the alternative pathway of complement activation^56^. Neutrophils can bind C3b to their surface and stabilize it via properdin to further enhance complement activation^57^. In turn, activated complement factor C5a actin induces neutrophil cytoskeleton polymerization and reorganization, resulting in increased membrane elasticity and cell size, enabling enhanced neutrophil migration and phagocytosis^58^. Therefore, antigen-specific IgG may initiate the classical complement pathway, with neutrophils sustaining complement activation through the alternative pathway. Activated complement factors could enhance neutrophil function, all of which may be necessary for killing *O. volvulus* L3.

The data presented here describe a mechanism by which vaccination results in the killing of a multicellular parasite through the collaboration of innate and adaptive arms of the immune response. The mechanism described in the present study does not kill *O. volvulus* larvae that survive in the host longer than 36 h and up to 3 weeks post challenge. This phenomenon may be due to some immune evasion mechanism of *O. volvulus*, like what has been described in primary infection with *Nippostrongylus brasiliensis*, where infective larvae can evade complement-mediated immunity within 24 h of infection^59^. Despite the ability of *O. volvulus* larvae to avoid killing within 24 h, they may develop susceptibility to the immune response again when the antigens *Ov*-103 and *Ov*-RAL-2 are expressed during the adult and microfilarial stages^17,22^. While we demonstrate one mechanism of killing in BALB/cByJ and C57BL/6J mice induced by *Ov*-FUS-1/Advax-CpG, other mechanisms may redundantly protect against *O. volvulus* challenge, as it has been previously shown that immunity induced by this vaccine can be polyfunctional in mice with diverse genetic backgrounds^19^.

The results of the present study identify a multi-component mechanism of vaccine-induced protective immunity, in which the innate and adaptive immune systems collaborate in stage-specific killing of *O. volvulus* L3. Antigen-specific IgG and enhanced neutrophil gene expression distinguished the effective immune response in immunized mice despite the ancillary modulation of neutrophil transcription by challenge larvae. The level of protection induced by the *Ov*-FUS-1/Advax-CpG vaccine, described here and previously^21^, has been predicted to be sufficient for controlling onchocerciasis and supports progress toward a Phase 1 clinical trial^12^. The required components for vaccine-induced protection identified in these studies will assist in monitoring vaccine efficacy and safety in humans and may be leveraged to understand how antigen and adjuvant combinations contribute to developing effective vaccines.

## Methods

### Source of parasites

*O. volvulus* L3 were collected from newly emerged adult *Simulium damnosum* black flies after feeding on consenting infected donors (Protocol 320, approved by the New York Blood Center and the Medical Research Station, Kumba, Cameroon, Institutional Review Boards). *S. damnosum* were housed in a controlled insectary and dissected after one week to collect, clean, and cryopreserve the L3, as previously described^60^.

### Source of mice

Six- to eight-week-old male BALB/cByJ, C57BL/6J, and B6.129S4-*C3*^*tm1Crr*^/J (C3^–/–^) mice were acquired from The Jackson Laboratory (Bar Harbor, ME, USA). B6.*Fcrα*^tm1tm2Rav^ /B6.*Fcgr1*^tm1Hoga^ (FcRα-null) mice were acquired from the laboratory of Jeffery V. Ravetch, M.D., Ph.D. at The Rockefeller University (New York, New York, USA)^30^. Mice were maintained in the Thomas Jefferson University Laboratory Animal Sciences Facility and housed in micro-isolator boxes in specific pathogen-free rooms under temperature, humidity, and light cycle-controlled conditions. Water and autoclaved rodent chow were provided to mice *ad libitum* (Philadelphia, PA, USA).

### Animal ethics

The animal use protocol (00136) was approved by the Thomas Jefferson University Institutional Animal Care and Use Committee (IACUC). Protocols and procedures were conducted in compliance with the ethical and regulatory standards for animal experimentation set by the National Institute of Health (NIH). All animal use protocols adhered to the “Guide for the Care and Use of Laboratory Animals” published by the National Research Council, USA.

### Anesthesia and euthanasia

For all procedures, mice were anesthetized with isoflurane, 3–5% induction and 1–3% maintenance (Aspen Veterinary Resources, Liberty, MO, USA). At the termination of each experiment, mice were euthanized under anesthesia by exsanguination for blood collection.

### Production of the recombinant fusion antigen

*Ov-*FUS-1 protein was designed, produced, and purified as previously described^21^. Briefly, *Ov*-FUS-1 was designed as a fusion of the open reading frames of the proteins *Ov-*103 and *Ov-*RAL-2, separated by a 15-amino acid glycine-serine linker comprised of the sequence GGGGSGGGGSGGGGS[(G4S)3], cloned into the vector pPICZ alpha (Thermo Fisher, Waltham, MA), and expressed in *Pichia pastoris* X-33 (Thermo Fisher). Production of *Ov*-FUS-1 was conducted in an Applikon ez-Control Bioprocessor (Applikon Biotechnology Inc., Foster City, CA, USA) using a clone of *P. pastoris* with high expression of *Ov*-FUS-1. Purification of *Ov-*FUS-1 protein occurred on an ÄKTA Pure Chromatography system (Global Life Sciences Solutions USA LLC, Marlborough, MA, USA). Purified protein was filter-sterilized through a 0.22 µm filter and stored at −80 °C. The purity and identity of the antigen were evaluated using three 1 μg aliquots of *Ov*-FUS-1 and bovine serum albumin (BSA) standard, were separated on a 4–20% Tris-glycine SDS-PAGE gel under reducing conditions and analyzed using ImageJ densitometry analysis software (https://imagej.net/ij/index.html) to confirm both the concentration and purity. Specificity was confirmed by Western blot analysis using specific murine *Ov*-FUS-1 antisera.

### Mouse immunizations

The *O. volvulus* vaccine was formulated using 50 µg of recombinant *Ov*-FUS-1 protein and 1 mg Advax-CpG (Vaxine Pty Ltd, Adelaide, Australia) containing the combination of 1 mg delta inulin and 10 µg CpG55.2, brought to a total volume of 100 µL using Tris-buffered saline (TBS) (Corning, Corning, NY, USA). Adjuvant-only control injections were formulated using 1 mg Advax-CpG, brought to a total volume of 100 µL with TBS. Injections were administered to anesthetized mice as 100 µL doses split into bilateral injections of 50 µL into each caudal thigh muscle. Mice were immunized on day 0, followed by booster injections on days 14 and 28.

### Diffusion chamber construction

Diffusion chambers were constructed using two different methods: For experiments in C3^–/–^ mice and those investigating the timepoint of larval killing, diffusion chambers were assembled out of 14 mm Lucite rings as previously described^17,21^. For all the remaining experiments, 3D-printed diffusion chambers (3Dp-DCs) were replicated using the previous standard diffusion chamber, fabricated using BioMed clear V1 resin.

Using computer-aided design SolidWorks Edition 2022–2023 (Dassault Systèmes, Waltham, MA), the 3D printed diffusion chamber (3Dp-DC) was created (Supplementary Fig. 4). Modifications were made to optimize the 3Dp-DC for printability and assembly. The 3Dp-DC consisted of one core (Supplementary Fig. 4a) and two caps (Supplementary Fig. 4b) on either side as one set (Supplementary Fig. 4c). CAD modifications included: (1) creation of a 10.15 mm diameter by 2.0 mm height cylinder (core) with a 2.7 mm wall to replicate the previous diffusion chambers; (2) Boolean subtraction of a 1.235 mm diameter to best plug the hole from one side of the core used to inject the *O. volvulus* L3 into the middle chamber; (3) creation of 0.6 mm thin caps with 10.15 mm diameter opening on either side of the core to make chamber assembly easier, diffusion chamber more stable, and reduce the glue from interacting with the mouse’s environment (Supplementary Fig. 4b); and (4) Boolean subtraction of the caps’ surface from the core, with a tolerance of 0.15 mm (Supplementary Fig. 4a) to ensure proper fit. The cap and core files were exported as surface tessellation language (STL) files for 3D printing.

The chambers were 3D printed in FormLabs BioMed clear V1 resin using stereolithography (SLA) on a FormLabs Form 3B printer (www.formlabs.com). SLA is an additive manufacturing type that uses resin to print highly accurate, watertight, isotropic products in various materials. The SLA process includes a build plate, photosensitive resin, a tank, and a UV laser. During printing, the build plate lowers into the liquid resin while a UV laser cures the layer base of the print. The plate then rises, allowing the excess resin to drip back down into the tank before the plate again lowers into the liquid resin to add the next layer. This process repeats, layer by layer, for hundreds of layers until the final model is finished. SLA can be printed with selected layer heights of 25, 50, or 100 µm, providing better resolution products when compared to other widely available desktop 3D printing modalities, such as fused filament fabrication. Additionally, SLA is a cost-effective resin option. FormLabs’ BioMed clear V1, a USP Class VI certified material manufactured by FormLabs’ FDA- registered, ISO 13485 certified facility, was chosen because of its strength, low water absorbance, and biocompatibility application for mucosal membranes (>30 h) 71. FormLabs BioMed clear V1 is compatible with most sterilization methods, including ethylene oxide.

The STL files of the chambers were prepared for 3D printing using Formlabs’ PreForm print preparation software (version 3.30.0). The 3Dp-DC cores were oriented perpendicular to the build plate for printing, with the injection hole oriented down towards the tank so that the uncured resin could drain from and drip off the print, returning uncured material to the tank. The 3Dp-DC caps were also oriented perpendicular to the build plate to maximize capacity and printing efficiency (Supplementary Fig. 4d). The density of supports was set to 0.7, touchpoint size was set to 0.45 mm, internal supports were turned off, full raft was turned on, and layer thickness was set to 50 µm. Excess supports were eliminated, so a minimal number of supports (6 for cores and 3 for caps) were used to pass the software’s print validation. Twenty-five sets (1 core + 2 membrane caps) were printed at once, requiring 68 ml of material and 5.5 printing hours.

The 3Dp-DCs were post-processed per the material manufacturer’s instructions. The 3Dp-DCs were washed in sterile isopropyl alcohol (IPA) in a FormLabs Wash for 15 min. The rafts were removed from the build plate using a spatula. The 3Dp-DC cores and caps were removed from the support structure and raft by clipping the vertical supports parallel to the cap/core.

Then, with a quick 90-degree twist, breaking the bottom support, the cap/core was freed from the raft. The irregular, remaining “nubs” left behind from broken supports were clipped with the clipper blades oriented parallel to the cap or core surface. Each cap and core was washed and rubbed with IPA to ensure any excess resin material was removed. The caps and cores were soaked in a fresh IPA wash for 5 min. The caps and cores were removed from the IPA wash, and each piece was allowed to air dry until dry to touch (minimum of 20 min) to ensure parts were dry and free of uncured resin. While drying, the injection holes in the cores were cleaned and ensured viable with an 18-gauge needle. The 3Dp-DC caps and cores were cured in the Formlabs Form Cure for 60 min at 60 °C per the instructions for use published by the material manufacturer. After curing, any residual support “nubs” were hand-sanded using an extra fine sanding block until the cap/core felt smooth and flush to the surface. This step ensured no sharp edges would unnecessarily irritate the mouse’s subcutaneous tissue.

After sanding, the 3Dp-DC caps and cores were “super cleaned” to ensure all excess particles were washed away before sterilization. A few drops of dish detergent were added to a water-filled sonicator. The caps and cores were placed directly in the sonicator basket and sonicated at 22 °C for 480 s. The caps and cores were removed from the sonicator and allowed to air dry for 20 min before packaging the chamber parts.

Final assembly of the diffusion chambers, constructed from either commercially produced Lucite rings or 3D printed rings, were adhered to 5.0 µm pore-size Durapore membranes (EMDMillipore, Burlington, MA, USA) to each side of the diffusion chambers^15,16,19,21^.

### Analysis of challenge infections

Cryopreserved *O. volvulus* L3 were processed as previously reported^15,16,19,21^. Twenty-five L3 were counted and loaded into each diffusion chamber and then surgically implanted in the subcutaneous space on the rear flank of each mouse while under anesthesia. The diffusion chambers were surgically removed from anesthetized mice at various time points, and the contents were collected for analysis. Larvae were considered to be alive if they displayed motility. The percent reduction in larval survival was calculated by [(mean count of surviving larvae from control animals – mean count of surviving larvae from immunized/treated animals) / mean count of surviving larvae from control animals] ×100. Parasites were removed from the diffusion chamber, and the remaining contents were centrifuged at 10,000 × *g* for 10 min at 22 °C. The supernatant was frozen for further analysis, and cells were counted using a hemocytometer (Reichert, Buffalo, NY, USA). Cells were applied to glass slides using a Cytospin 3 centrifuge (Shandon, Pittsburgh, PA, USA) and stained using a Hema 3 staining kit (Thermo Fisher Scientific, Waltham, MA, USA) following the manufacturer’s protocol.

### Serum collection and IgG purification

Mice were immunized with *Ov*-FUS-1/Advax-CpG following the protocol described above. Two weeks post-final-booster immunization, blood was collected from immunized or age-matched naïve mice while under anesthesia by exsanguination and left to clot at 4 °C for 1 h. Clotted blood was centrifuged at 10,000 × *g* for 10 min at 22 °C. The serum layer was removed and aliquoted into 1.5 mL Eppendorf tubes and stored at –80 °C until later use.

IgG was purified from serum that was collected from vaccinated mice. Pooled immune serum was thawed, and NaPO_4_ (FisherScientific) was added to a final concentration of 20 mM. Precipitate was removed by centrifugation of the serum with NaPO_4_ at 50,000 × *g* for 5 min at 4 °C, and the supernatant was collected. Purification was conducted by passing the serum with NaPO_4_ over a 5 mL HiTrap Protein G HP column (Cytiva, Marlborough, MA, USA) attached to an ÄKTAprime plus chromatography system (Cytiva) following the manufacturer’s protocol. IgG was bound to the column in 20 nM NaPO_4_ with 0.1 M EDTA (pH 7). The 1 mL IgG fractions were eluted by passing 0.1 M glycine-HCl (pH 2.7) over the column. Purified IgG was eluted into tubes containing 130 μL of 1 M Tris-HCl (pH 9) and immediately moved to ice. Eluted IgG fractions were pooled and dialyzed by loading into 6000–8000 molecular weight cut-off (MWCO) dialysis tubing (Fisher) suspended in 6 L PBS for 48 h at 4 °C. Dialyzed IgG was then concentrated 15 mL at a time by centrifuging in 30,000 MWCO centrifuge filter tubes (EMD Millipore) at 3200 × *g* for 30 min at 25 °C^32^. The concentration of the purified IgG was determined by ELISA, and the concentrate was filter sterilized and stored at –80 °C^32^.

### Passive transfer of immunity

Pooled serum from naïve or immunized mice, or purified immune IgG, was filter sterilized. Purified immune IgG was diluted with TBS to a concentration of 1.5 mg/mL to match the IgG concentration of the pooled immune sera. Mice were challenged with L3 in diffusion chambers, and 100 μL of serum or purified IgG were injected subcutaneously adjacent to the diffusion chamber at the time of challenge. A second dose was administered three days post-challenge at the same location while mice were under anesthesia. The diffusion chambers were removed seven days post-challenge, and the contents were collected for analysis.

### RNA sequencing of diffusion chamber cells

Cells from diffusion chambers recovered 18 and 36 h post challenge were isolated by centrifugation at 10,000 × *g* for 10 min at 20 °C. The total RNA was isolated using the Qiagen RNAeasy Plus kit. RNA concentration was quantified using a Qubit fluorimeter, and integrity was determined by the TapeStation high-sensitivity RNA kit (Agilent Technologies, Santa Clara, CA, USA). All samples had RNA integrity numbers (RIN) greater than 7. Next, mRNA libraries were prepared by poly-A selection from 1 ng of each sample using the Takara SMART-seq HT kit (Takara, 634437). Libraries were sequenced on the NextSeq 2000 (Illumina) sequencer, using 100-cycle single-read settings. At least 18 million reads were obtained per sample. Using a Linux computing cluster, sequencing reads were trimmed to remove low-quality or adapter-containing sequences using Trimmomatic v. 0.39^61^, with the following settings LEADING:3 TRAILING:3 SLIDINGWINDOW:4:15 MINLEN:36. Trimmed reads were mapped to the NCBI mm10 *Mus musculus* reference genome using STAR^62^. Mapped reads were quantified using HTseq-count, with the following settings --mode union -a 1. Eleven to 30 million reads mapped uniquely to the reference genome for each sample^63^. Mapped read count data were further analyzed in R, as follows: Counts were filtered to retain genes with count values greater than 0 for six or more samples, normalized and scaled to obtain counts per million (CPM) values using edgeR^64^, which were used for hierarchical clustering of Euclidian distances, computed in R. Differentially expressed genes between immunized versus control groups were identified within each time point (18 h or 36 h), for diffusion chambers with or without larvae, using DESeq2^65^. For each grouping (i.e., 18 h with larvae, 18 h without larvae, 36 h with larvae, 36 h without larvae), a DESeq2 model was fit using ~immunization as the model design parameter. Volcano plots were generated using ggplot in R. GSEA was performed using the GSEA software on normalized expression values for each sample grouping. The hallmarks gene set database was acquired from the MSigDB 7.0^25^. Lists of neutrophil subset signature genes were compiled based on the expression profiles of prior studies^26,66^.

### Depletion of cells and complement

To deplete granulocytes, the monoclonal antibody (MAb) RB6-8C5, rat anti-Gr1, was used (Bio X Cell, Lebanon, NH, USA). Mice were given an intraperitoneal injection of 0.5 mg diluted in *InVivo*Pure pH 7.0 Dilution Buffer (Bio X Cell) three days before and at the time of the challenge. Control mice received the isotype control rat IgG2b isotype control (clone: LTF-2) (Bio X Cell). The depletion of granulocytes was confirmed by microscopic analysis of diffusion chamber cells, which were differentiated based on morphology. Cobra venom factor (CVF) from the lyophilized venom of *Naja kaothia* (Complement Technology, Tyler, TX, USA) was used to deplete complement. Mice received an intraperitoneal injection of 400 µg/kg CVF dose in sterile PBS, or PBS only, one day before and at the time of challenge.

### Enzyme-linked immunosorbent assay analysis of complement components and neutrophil granular products

Diffusion chamber supernatants were thawed, and the concentration of C3, complement component C3a (C3a), neutrophil elastase (NE), and myeloperoxidase (MPO) were measured by ELISA. The Mouse Simple Step ELISA Kit (Abcam, Waltham, MA, USA) was used to analyze C3, MPO, and NE. Final sample dilutions were: C3: 1:100,000, NE: 1:4000, and MPO: 1:12.5, whereas C3a was measured using the Mouse Complement Component 3a ELISA Kit (Invitrogen, Waltham, MA, USA), with a final sample dilution of 1:25,000. All kits were used following manufacturers’ protocols, and optical densities were measured at 450 nm using an iMark plate reader (BioRad, Hercules, CA, USA) with SoftMax Pro version 6.5.1 software (Molecular Devices, San Jose, CA, USA).

### Immunofluorescence microscopy

*O. volvulus* L3 were thawed, aliquoted into Eppendorf tubes, fixed in 2% paraformaldehyde (Electron Microscopy Sciences, Hatfield, PA, USA) for 20 min at 22 °C, and then washed. All washes were conducted with 10% goat serum (Fisher Scientific) in PBS by centrifugation at 3000 × *g* for 4 min at 22 °C. All incubation steps were for 1 h at 4 °C. Samples were blocked in 1 mL of 10% goat serum. Each tube was incubated in 100 µL volumes of 1.5 μg of either naïve IgG from mouse serum (Sigma) or IgG purified from *Ov*-FUS-1/Advax-CpG immunized BALB/cByJ mice in wash buffer. After washing, the larvae were stained with either (1) 1 μL goat anti-mouse IgG [AlexaFluor 488] or (2) 1 μL of goat anti-mouse IgG1 [AlexaFluor 488], 1 μL goat anti-mouse IgG2a [AlexaFluor 555] and 1 μL goat anti-mouse IgG2b [AlexaFluor555] (Invitrogen). After the final wash, each sample was resuspended in 100 μL of 10% goat serum, and the larvae were mounted onto Colorfrost slides (Fisher) by centrifugation with a Cytospin 3 centrifuge. Glass coverslips of 1.5 thickness were applied with ProLong glass antifade mountant (Invitrogen) and left to cure overnight in the dark.

Imaging of stained *O. volvulus* L3 was conducted on a Nikon A1R point-scanning laser confocal microscope (Nikon, Minato City, Tokyo, Japan). Images were analyzed by generating sum-of-slice Z projections and calculating the mean gray values over the area of each larva using the Fiji suite of plugins for ImageJ (https://imagej.net/software/fiji/, version 2.16.0)^67,68^.

### Statistics

Mouse experiments consisted of 5–6 mice per group and were repeated two times with consistent results between experiments. Data from repeated experiments were combined for statistical analyses where appropriate. Differences between larval survival and differentiated cell counts in diffusion chambers were determined by multi-factorial analysis of variance with post-hoc Fisher’s least significant difference testing in Systat v.11 (Systat Inc., Evanston, IL, USA). Probability values (*p*) of ≤ 0.05 were considered statistically significant.

## Supplementary information

Supplemental Figures and Tables.

## Data Availability

The data that support the findings of this study are available in the article and in the supplementary figures, tables and information. The mRNA sequence data generated an analyzed for the present study are available within the National Center for Biotechnology Information's Gene Expression Omnibus under the accession number GSE295728 (https://www.ncbi.nlm.nih.gov/geo/query/acc.cgi?acc=GSE295728).
