# Supplementary material for: Vaccination against Onchocerca volvulus induces IgG-mediated protective immunity dependent on neutrophils and complement
Source: NPJ Vaccines. 2025 Oct 22;10:221. doi: 10.1038/s41541-025-01267-x (PMC12546713; doi:10.1038/s41541-025-01267-x)
Supplement: Supplementary file 1 — Supplemental Figures and Tables. [file 41541_2025_1267_MOESM1_ESM.pdf]

SUPPLEMENTARY FIGURES AND TABLES

**Recovery time point**	**18 hours post-challenge**			
**Immunization**	**Control**		**Immune**	
**Challenge**	+L3	-L3	+L3	-L3
Total cells (x10^6)	5.35 ± 2.46	7.59 ± 2.50	3.73 ± 1.36	4.23 ± 2.44
Neutrophils	98% ± 1%	99% ± 1%	99% ± 1%	98% ± 2%
Macrophages	0% ± 1%	1% ± 1%	0% ± 0%	1% ± 1%
Eosinophils	1% ± 2%	0% ± 1%	1% ± 1%	1% ± 1%
**Recovery time point**	**36 hours post-challenge**			
**Immunization**	**Control**		**Immune**	
**Challenge**	+L3	-L3	+L3	-L3
Total cells (x10^6)	3.88 ± 2.73	5.92 ± 2.47	5.33 ± 2.73	6.35 ± 4.13
Neutrophils	94% ± 4%	96% ± 3%	96% ± 4%	95% ± 3%
Macrophages	1% ± 1%	1% ± 2%	1% ± 2%	0% ± 1%
Eosinophils	5% ± 4%	3% ± 2%	5% ± 3%	5% ± 3%

Supplementary Table 1. Diffusion chamber cells from immunized mice measured 18 and 36 hours post-challenge with or without *O. volvulus* L3. BALB/cByJ mice were immunized with *Ov*-FUS-1/Advax-CpG and challenged with diffusion chambers containing either *O. volvulus* L3 (+L3) or media only (-L3). Recovery of diffusion chambers occurred 18 or 36 hours post-challenge. Cells within the diffusion chambers were counted and differentiated morphologically. Data represent mean total cell counts and percentages of neutrophils, macrophages, and eosinophils (± standard deviations).

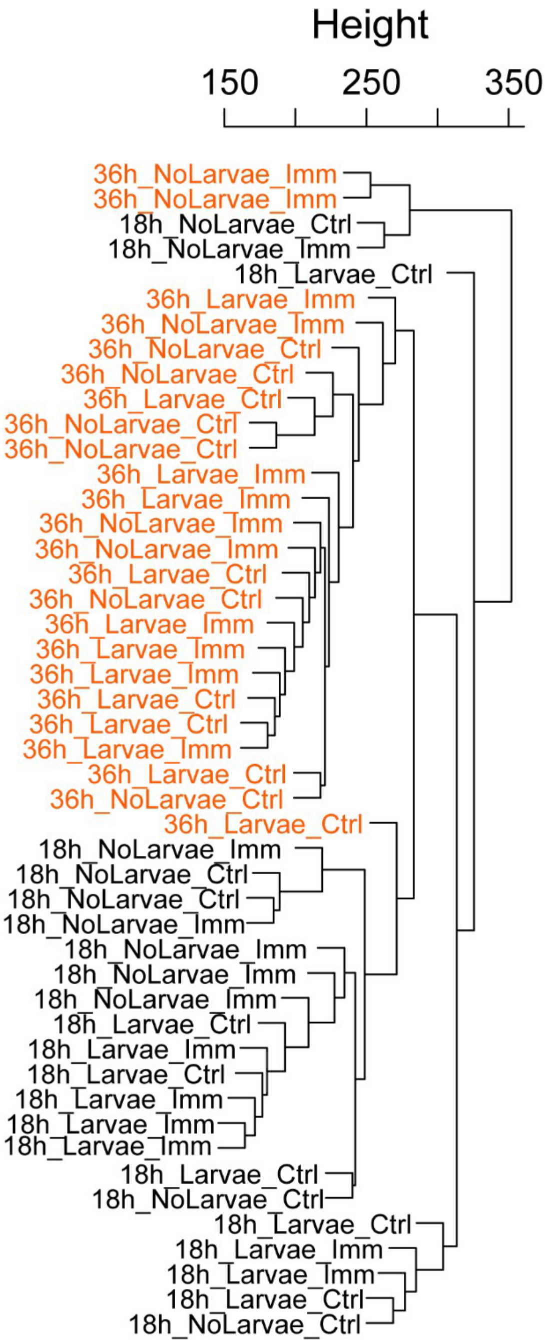

Supplementary Figure 1. Hierarchical clustering of Euclidian distances. BALB/cByJ mice were immunized and challenged. Recovery of diffusion chambers occurred 18 and 36 hours post-challenge, and cells were collected for bulk RNA-Seq. Data represents hierarchical clustering of Euclidian distances based on total gene expression. Black-colored sample names indicate 18 hours post-challenge, with orange indicating 36 hours post-challenge.

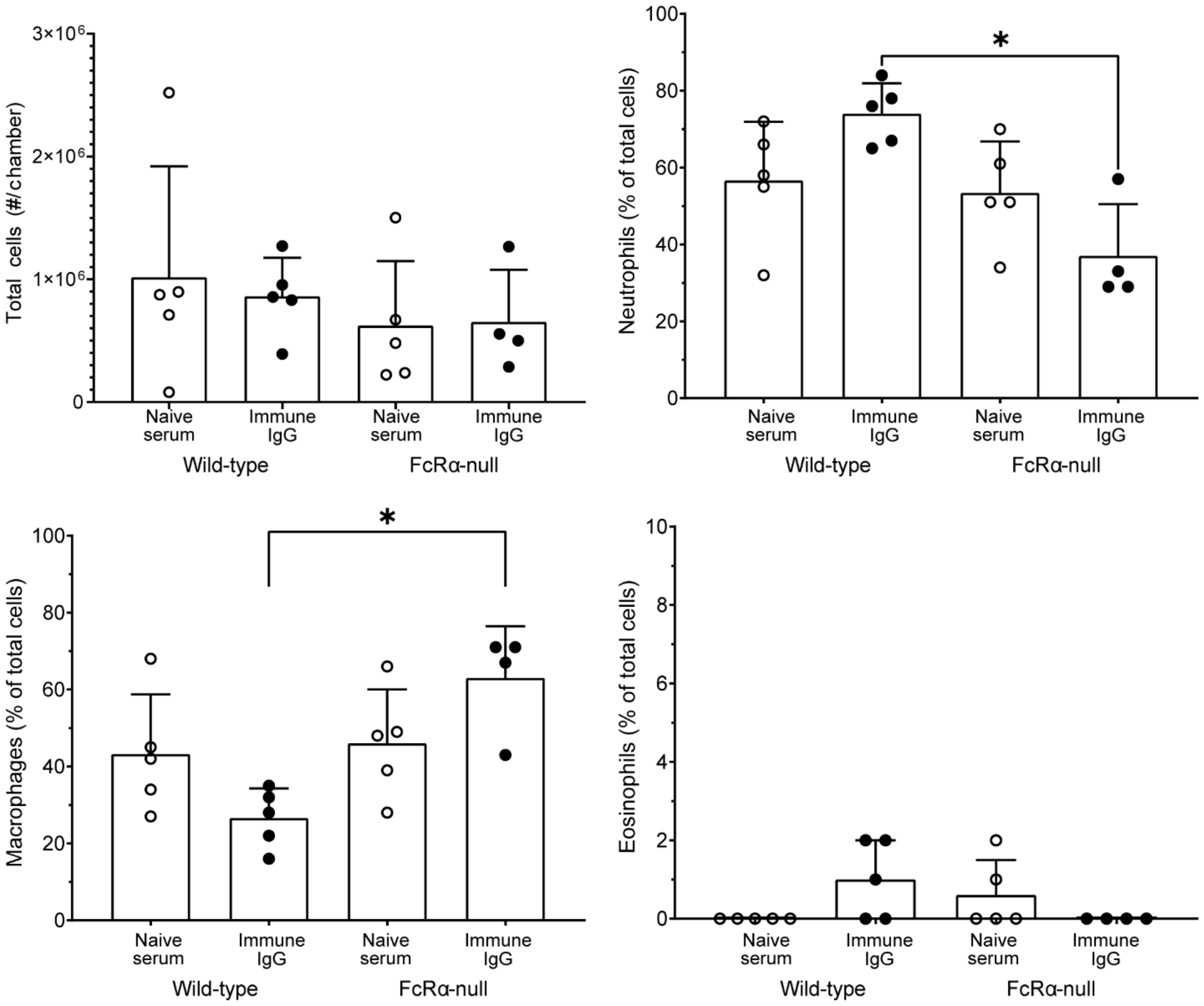

Supplementary Figure 2. Diffusion chamber cells from purified immune IgG-treated FcγR-deficient mice. C57BL/6J wild-type and FcRα-null mice received either naïve serum or purified immune IgG. Total cells and differential cell counts of neutrophils, macrophages, and eosinophils as a percentage of total cells in diffusion chambers recovered seven days post-challenge. Data are shown as mean total cell counts or percentage of total cells, with points representing individual mice and error bars representing standard deviations. * = p value < 0.05, indicating statistically significant differences when comparing groups.

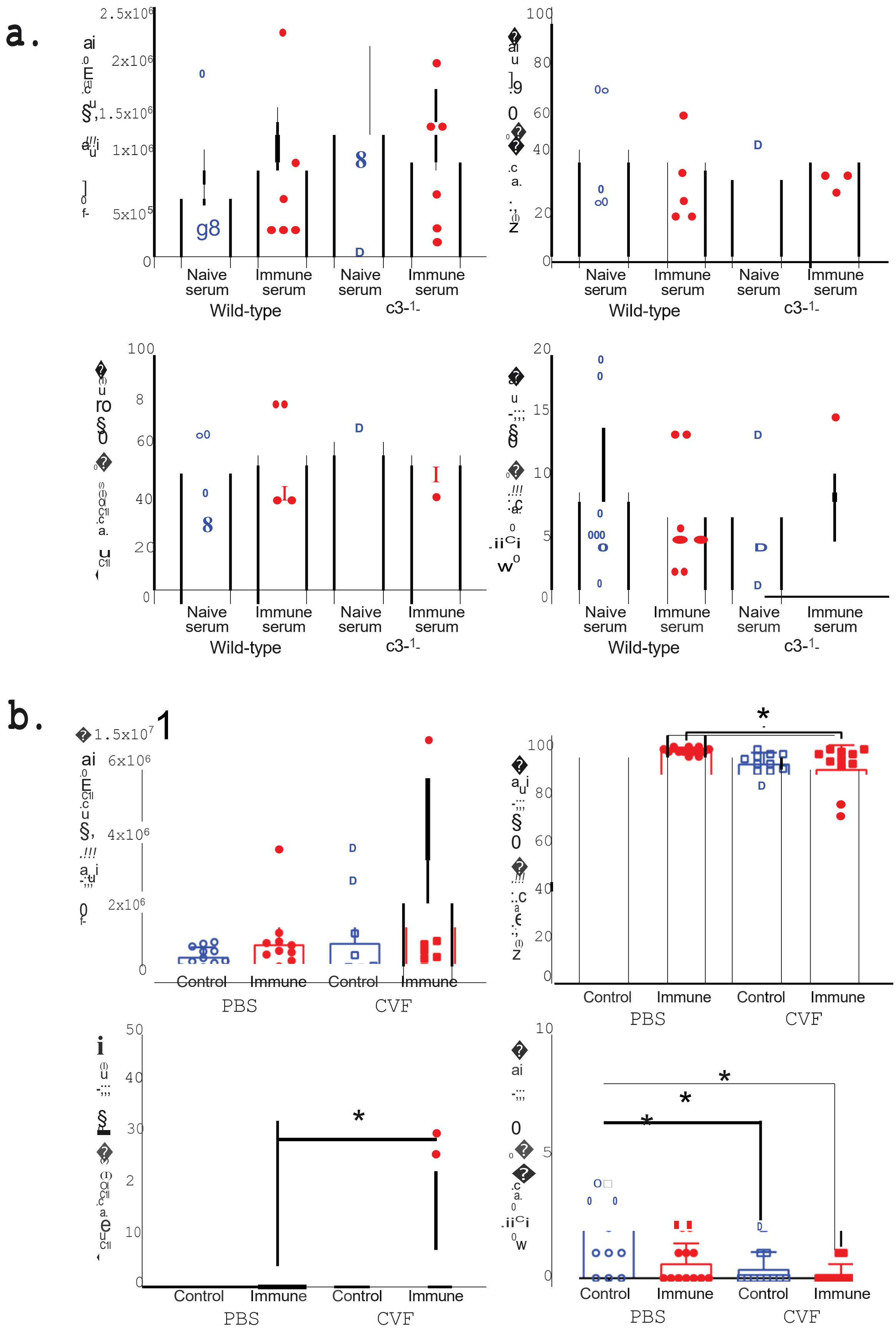
a.
b.
Naive serum
Immune serum
Wild-type
Control
Immune
PBS
CVF
2.5x10^6
2x10^6
1.5x10^6
1x10^6
5x10^5
1.5x10^7
6x10^6
4x10^6
2x10^6
100
80
60
40
20
50
10
5
*

Supplementary Figure 3. Diffusion chamber cells from immune serum-treated C3-deficient and vaccinated C3-depleted mice. (**a**) C57BL/6J wild-type and $C3^{-/-}$ mice were challenged and treated with either naïve or immune serum. (**b**) BALB/cByJ mice were immunized, challenged, and treated with either CVF or PBS control. Data presented are total cell counts and percentage of neutrophils, macrophages, and eosinophils measured within diffusion chambers recovered at seven days (**a**) or 48 hours (**b**) post-challenge. Data points represent measurements from individual mice, and error bars represent standard deviations. * = p value < 0.05, indicating statistically significant differences when comparing groups.

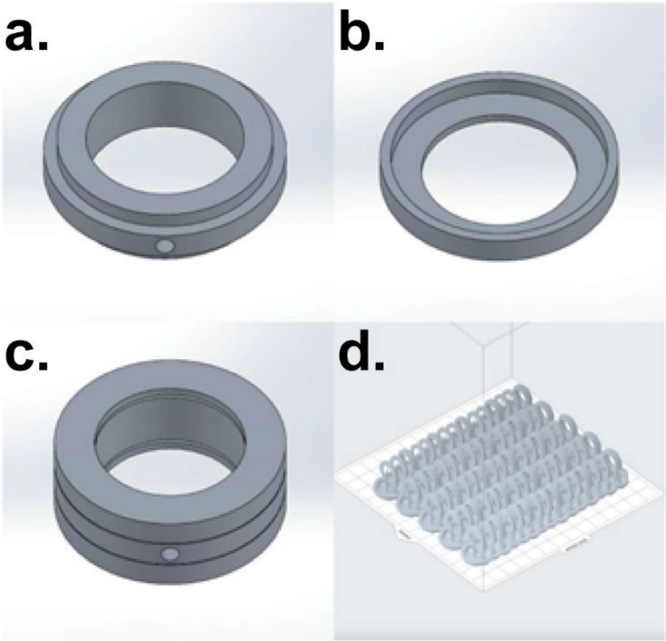

Supplementary Figure 4. 3D printed diffusion chamber schematic. SolidWorks' 3D virtual model of 3Dp-DC's (**a**) core, (**b**) single cap, and (**c**) complete set. (**d**) Twenty-five sets of 3Dp-DCs plated in Formlabs' PreForm print preparation software.

SUPPLEMENTAL INFORMATION

For the purpose of initial peer review supplementary files and the RNAseq data set can be accessed following the provided link (https://jefferson.box.com/s/zqlfg5oy1gi09mcbept9e7b8e7j36ybc). Files are in the process of being uploaded to the Gene Expression Omnibus (GEO).
